# Current concepts on endothelial stem cells definition, location, and markers

**DOI:** 10.1002/sctm.21-0022

**Published:** 2021-11-01

**Authors:** Sarah E.J. Chambers, Varun Pathak, Edoardo Pedrini, Lou Soret, Nicolas Gendron, Coralie L. Guerin, Alan W. Stitt, David M. Smadja, Reinhold J. Medina

**Affiliations:** ^1^ Wellcome‐Wolfson Institute for Experimental Medicine, School of Medicine, Dentistry, and Biomedical Science, Queen's University Belfast Belfast UK; ^2^ Université de Paris Innovative Therapies in Haemostasis, INSERM Paris France; ^3^ Hematology department and Biosurgical research lab (Carpentier Foundation) Assistance Publique Hôpitaux de Paris.Centre‐Université de Paris (APHP‐CUP) Paris France; ^4^ Cytometry Platform, Institut Curie Paris France

**Keywords:** angiogenesis, blood vessels, circulating endothelial progenitor cells, endothelial colony‐forming cells, endothelial stem cells, neovascularization, vascular repair

## Abstract

Ischemic vascular disease is a major cause of mortality and morbidity worldwide, and regeneration of blood vessels in perfusion‐deficient tissues is a worthwhile therapeutic goal. The idea of delivering endothelial stem/progenitor cells to repair damaged vasculature, reperfuse hypoxic tissue, prevent cell death, and consequently diminish tissue inflammation and fibrosis has a strong scientific basis and clinical value. Various labs have proposed endothelial stem/progenitor cell candidates. This has created confusion, as there are profound differences between these cell definitions based on isolation methodology, characterization, and reparative biology. Here, a stricter definition based on stem cell biology principles is proposed. Although preclinical studies have often been promising, results from clinical trials have been highly contradictory and served to highlight multiple challenges associated with disappointing therapeutic benefit. This article reviews recent accomplishments in the field and discusses current difficulties when developing endothelial stem cell therapies. Emerging evidence that disputes the classic view of the bone marrow as the source for these cells and supports the vascular wall as the niche for these tissue‐resident endothelial stem cells is considered. In addition, novel markers to identify endothelial stem cells, including CD157, EPCR, and CD31^low^ VEGFR2^low^ IL33^+^ Sox9^+^, are described.

## Graphical abstract

Understanding endothelial stem/progenitor cells.

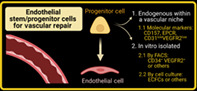

**Significance statement**

The present study provides a scientific update on recent advances in endothelial stem cell definition, location, and markers. Current challenges for an effective translation of cellular therapies for vascular repair and regeneration are discussed, and potential strategies to overcome these challenges are proposed. The importance of a molecular marker profile combined with functional assays when defining endothelial stem/progenitor cells is highlighted.

## 1 DEFINING ENDOTHELIAL STEM CELLS

Development studies in mice have established the origin of blood vessel formation to occur with the appearance of angioblast precursors derived from early mesoderm, which leads to the formation of the primitive vascular network within the extraembryonic yolk sac at E6.5.^1^ These angioblasts that form blood islands also migrate to colonize embryonic tissues. During subsequent vasculogenesis, extensive remodeling of the primitive vasculature leads to formation of a functional vascular network consisting of specialized arterial, venous, lymphatic, and hemogenic endothelial cell subtypes. Colonization of the bone marrow occurs later by hematopoietic stem cells arising from the hemogenic endothelium at E16.5.^2^ The close physical, temporal, and spatial association between hematopoietic and endothelial cells during embryonic development has given rise to theories such as the existence of a common bipotent progenitor known as the hemangioblast. While the existence of the hemangioblast is still debated,^3^ this relationship between endothelial and hematopoietic cells is also shown in the hemogenic endothelium, which has been defined as a specialized endothelial cell population that gives rise to hematopoietic cells. The shared expression of markers, such as CD34 and VEGFR‐2 (in human); or sca1 and flk‐1 (in mouse), in cells with capacity for hematopoietic and endothelial differentiation led to the proposal for an adult hemangioblast circulating in peripheral blood.^4^ These cells have been studied as putative circulating endothelial progenitor cells (EPCs) with vasoreparative properties. While in preclinical models, transplantation of these EPCs promoted angiogenesis in ischemic tissues,^5^ follow on clinical trials have not shown similar efficacy.^6^ There is significant controversy defining EPCs, as the term has been broadly used to describe cells that are non‐endothelial in nature, such as hematopoietic stem cells (HSCs) and myeloid angiogenic cells (MACs).^7^ Furthermore, studies have demonstrated that blood monocytic cells can acquire endothelial markers in culture by uptake of platelet extracellular vesicles.^8^

Following basic stem cell biology principles, endothelial stem cells must exhibit capacities for self‐renewal and lineage differentiation into organ‐specific specialized endothelial cells. Evidence has confirmed a higher cell turnover rate in adult cardiac tissue for endothelial cells (>15% per year), when compared with mesenchymal cells (<4% per year) or cardiomyocytes (<1% per year).^9^ Similarly, multi‐isotope imaging mass spectroscopy and electron microscopy has revealed significant endothelial cell turnover in brain, liver, and pancreas, with blood vessels being described as mosaics structures consisting of young and aged cells.^10^ Furthermore, in the adult bone marrow, a small population of Apelin expressing endothelial cells is critical for vascular regeneration after irradiation.^11^ All these data suggest that endothelial homeostasis and maintenance is preserved by a self‐renewing subpopulation of cells with stem/progenitor properties. During development, initially homogeneous embryonic endothelial precursors acquire distinct identities to support organ relevant function. This endothelial cell heterogeneity in different tissues and organs have been described at the transcriptome and translatome level.^12^, ^13^ In addition, endothelial cells can adopt tissue‐specific characteristics during organ development and regeneration.^14^ This endothelial specialization according to organ microenvironment underscores the plasticity of some endothelial cells, which also suggests the existence of an endothelial stem cell. Hematopoietic stem cells are the quintessential example for a precise stem cell definition, not only by molecular markers but with robust evidence for lineage differentiation and self‐renewal that goes beyond the lifespan of the HSC donor.^15^ This scientific stringency is currently missing when defining endothelial stem cells. Therefore, we propose that an endothelial molecular identity coupled with self‐renewal and differentiation capacity into various endothelial cell phenotypes should be essential requirements to define endothelial stem cells. While the terms endothelial stem and progenitor are currently used interchangeably, future studies are needed to formulate models for endothelial differentiation hierarchy and distinguish stem from progenitor cells. Until evidence for precise molecular identities is established, we hypothesize that endothelial stem cells must retain the capacity to differentiate into arterial, venous, lymphatic, and capillary endothelial cells as required. This can be demonstrated by their molecular plasticity and adoption of organ specific endothelial characteristics, akin to induced pluripotent‐derived endothelial cells.^14^ Endothelial progenitors, on the other hand, are expected to be committed to targeted differentiation into organ specialized endothelium, suggesting that an endothelial progenitor cell niche exists within highly vascularized organs.

Endothelial colony‐forming cells (ECFCs) are a subtype of endothelial progenitor with remarkable clonogenic potential and postnatal vascularization ability in vivo.^16^ ECFCs are consistently isolated from human cord blood and there is an established consensus for their definition.^17^ ECFCs have been shown to significantly contribute to vascular regeneration of ischemic tissues such as heart, brain, retina, and limbs^18^; and they are on a translational pathway toward a cell therapy.^19^ A systematic review of controlled preclinical animal studies using human ECFCs concluded that while the potential clinical application for ECFCs is evolving rapidly, the wider implementation of already established standardized ECFC characterization will enable more rapid and effective transition to clinical trials.^20^

## 2 LOCATION OF ENDOTHELIAL PROGENITORS

EPCs were originally thought to reside in the bone marrow^21^; however, recent studies in patients with sex‐mismatched bone marrow transplantations have shown that these endothelial vasoreparative cells do not originate from bone marrow.^22^ ECFCs isolated from venous wall and peripheral blood of male patients, who had previously received bone marrow transplants from female donors, displayed a XY genotype, which negates bone marrow as the ECFC source. In agreement with the idea for a vascular niche, ECFCs have been isolated from human saphenous vein.^23^ Evaluation of transcriptomic and proteomic profiles in human peripheral blood‐derived ECFCs suggested that they represent an intermediate endothelial cell population between human coronary artery endothelial cells and human umbilical vein endothelial cells.^24^ While these studies suggests that ECFCs reside within macrovessels, these progenitors have also been isolated from microvessels of human placenta.^25^ Interestingly, microvascular ECFCs show greater vessel forming capacity than macrovascular ECFCs in a Matrigel graft implant model. Furthermore, there is evidence that ECFCs are also resident in white adipose tissue (WAT). ECFCs isolated from WAT stromal vascular fraction displayed comparable angiogenic properties to umbilical cord and peripheral blood‐derived ECFCs.^26^ Although evidence suggests ECFCs arise from a vascular niche, their origin and specific location as vascular wall resident endothelial cells in vivo remains to be proven.

**FIGURE 1:**
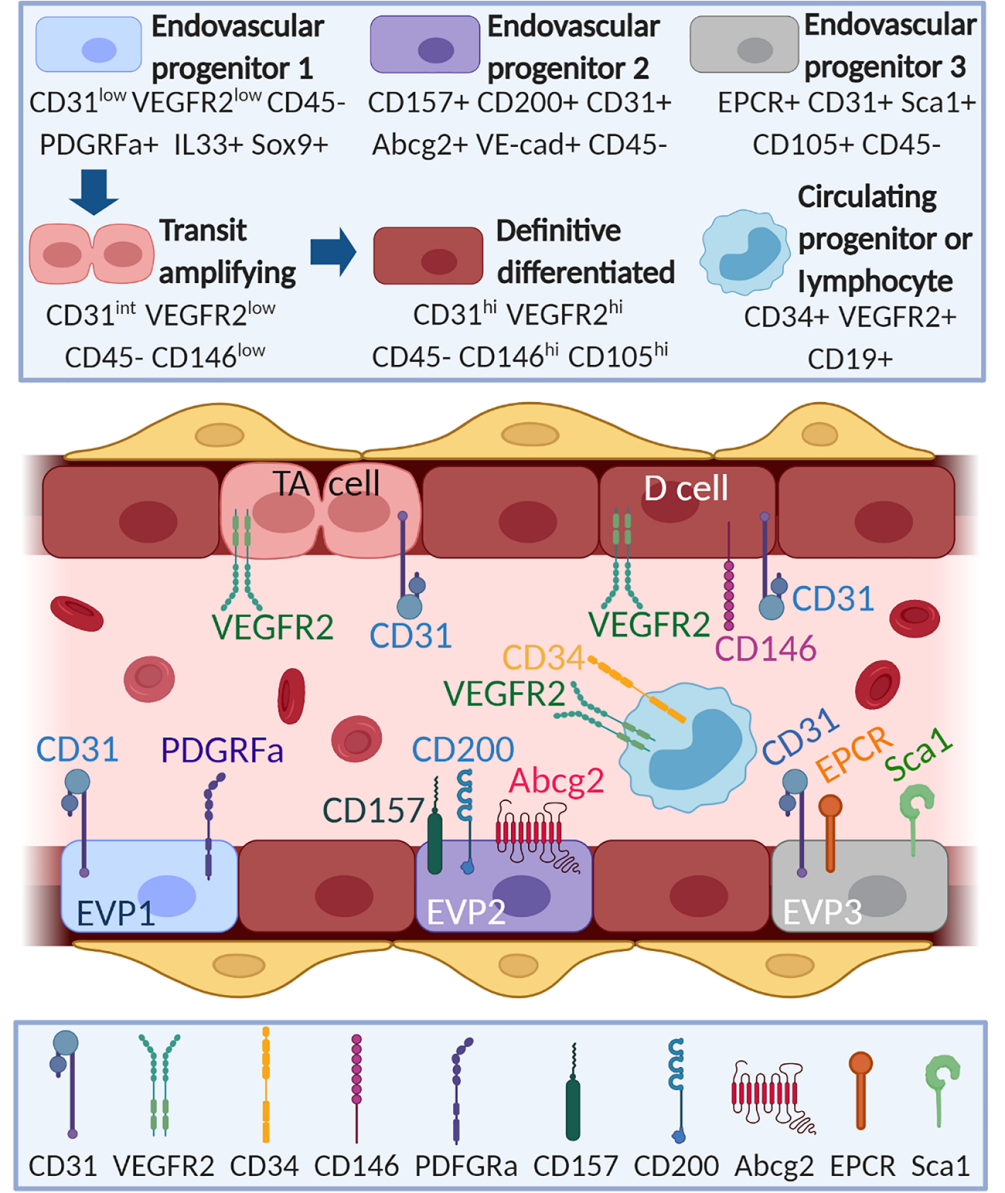
Molecular identities reported for endogenous endothelial stem/progenitor cells. Diagram to depict endovascular progenitor cell types that reside within the vasculature and have vasoreparative properties reported in mouse tissues. Cell types shown in upper box. Cell surface markers shown in lower box. D cell, definitive differentiated; EVP, endovascular progenitor; TA, transit amplifying. Figure created in BioRender.com

Murine models of vascular injury have enabled further understanding of the functional significance for vasoreparative cells within local vascular niches. Endothelial regeneration of pulmonary vasculature in an endotoxin‐induced lung injury model have suggested that tissue resident endothelial progenitors contribute to vascular repair of pulmonary capillary vessels which did not originate from bone marrow.^27^ Similarly, a study using an aortic injury model showed that regeneration of mouse aorta is executed by endothelial cells from the border regions of the vascular lesion.^28^ Endothelial cells flanking the lesion enter mitosis and local endothelial cell proliferation through transient amplifying cell populations is responsible for vascular wound closure without the significant contribution of bone marrow‐derived progenitors. Further lineage tracing experiments have revealed an endothelial hierarchy with three subsets: endovascular progenitors (EVP, CD31^low^ VEGFR2^−/low^), transient amplifying (TA, CD31^int^VEGFR2^−/low^), and definitive differentiated (D, CD31^high^VEGFR2^high^) cells (Figure 1). This study demonstrated that EVPs, but not TA or D cells, exhibited intrinsic stem/progenitor cell properties, including in vivo self‐renewal potential.^29^ Unbiased single‐cell RNA sequencing from mouse aortas confirmed this endothelial hierarchy, and identified Sox9, Il33, and PDGFRA as molecular markers for EVP, while CD31 and Sox18 identified differentiated cells.^30^ Similarly, highly proliferative and vasoreparative CD157^+^CD200^+^ cells were found in large adult murine vessels, which were also identified as tissue resident endothelial stem cells.^31^ Interestingly, EVPs highly expressed Pdgfra, a mesenchymal gene which indicates their potential to differentiate into endothelial and mesenchymal cell types. In agreement with this theory, genetic lineage tracing studies and single cell RNA sequencing in the murine model of hindlimb ischemia has shown that a subset of fibroblasts can acquire endothelial genes.^32^ The emergence of CD144^+^ cells within FSP (fibroblast‐specific protein)‐1+ fibroblasts in ischemic limbs was mediated by Toll‐like receptor‐3 and nuclear factor κB. On the contrary, in the mouse model of myocardial ischemia, lineage tracing experiments pointed that pre‐existing endothelial cells and not fibroblasts, were responsible for the observed neovascularization.^33^ These studies highlight the importance of fibroblasts in promoting vascularization, and further investigations are warranted to confirm the existence of a bipotent mesenchymal stem cell or fibroblast with capacity for mesenchymal to endothelial transition.

An unambiguous molecular definition of endothelial stem cells phenotype and status is lacking. However, based on current evidence, it can be considered that these stem cells lie dormant in healthy tissues, but with the capacity to rapidly respond by proliferating, differentiating into endothelium and thereby contributing to vascular homeostasis and repair. Whether they engage with repair by directly differentiating into endothelium or evoke a more complex pathway involving transient amplifying cells is unknown. Clearly further research is needed since the process of vascular repair is complex and expected to involve not only endothelial stem/progenitor cells but also differentiated endothelial cells, mural cells, immune cells, and platelets.

## 3 CLINICAL TRANSLATION CHALLENGES FOR ENDOTHELIAL STEM/PROGENITOR CELLS

Since the early 90s, there has been considerable interest in endothelial stem/progenitor cells as a potential cell therapy for ischemic diseases. Unfortunately, meta‐analysis of clinical trials for ischemic heart disease^34^ and critical limb ischemia,^6^ have indicated that while such cell therapies are safe, they have limited therapeutic efficacy. Among the factors contributing to these disappointing results are (a) Lack of accurate definitions for cell therapy products; (b) Absence of standardization on cell dosage or delivery route; (c) Patient target population with severe disease; (d) Poor cell survival and engraftment after delivery into hypoxic scarred tissue; and (e) Undefined mechanism of action. In summary, the rapid move of endothelial progenitor cell therapies from the labs into the clinics, without having well‐defined cells with a proven mechanism of action has negatively impacted the results. We propose some strategies to dispel these challenges (Table 1). Moreover, advances in stem cell characterization at single‐cell resolution alongside emergence of novel biomaterials will improve delivery of cell therapies using endothelial stem cells. A detailed understanding of distinct type of vasoregenerative cells has translational applications, by providing a scientific rationale to base clinical trial design and maximize therapeutic efficacy. Cumulative evidence has demonstrated that circulating CD34^+^VEGFR2^+^ cells, MACs, and ECFCs, all contribute to reparative angiogenesis by different mechanisms of action. Choosing the most appropriate cell type will depend on disease type. For example, if ischemic tissue retains endogenous vasoreparative capacity, MACs will provide the pro‐angiogenic support needed to enhance endothelial regeneration; however, if endothelial repair is significantly impaired such as in diabetic vasculopathies, then an allogeneic approach using ECFCs will facilitate effective de novo therapeutic angiogenesis.

**TABLE 1:** Analysis of challenges and potential solutions to address difficulties when developing endothelial cell therapies

Challenges for effective translation of endothelial stem/progenitor cell therapies	Proposed solutions
Unclear definitions for identity and purity of cells	Detailed release criteria based on cell surface immunophenotype and potency assays
2. Lack of standardization for cell dosage and delivery routes	Preclinical dose scalation and delivery route studies, to identify most effective strategy
3. Patient target population with severe disease	Phase II efficacy trials may require a patient population with a wider range of disease severity than Phase I safety trials
4. Poor cell survival and engraftment	New approaches that modulate the microenvironment and/or cells to increase resilience to hypoxia
5. Undefined mechanism of action	Preclinical data that defines mechanism of action at the molecular level is established

**FIGURE 2:**
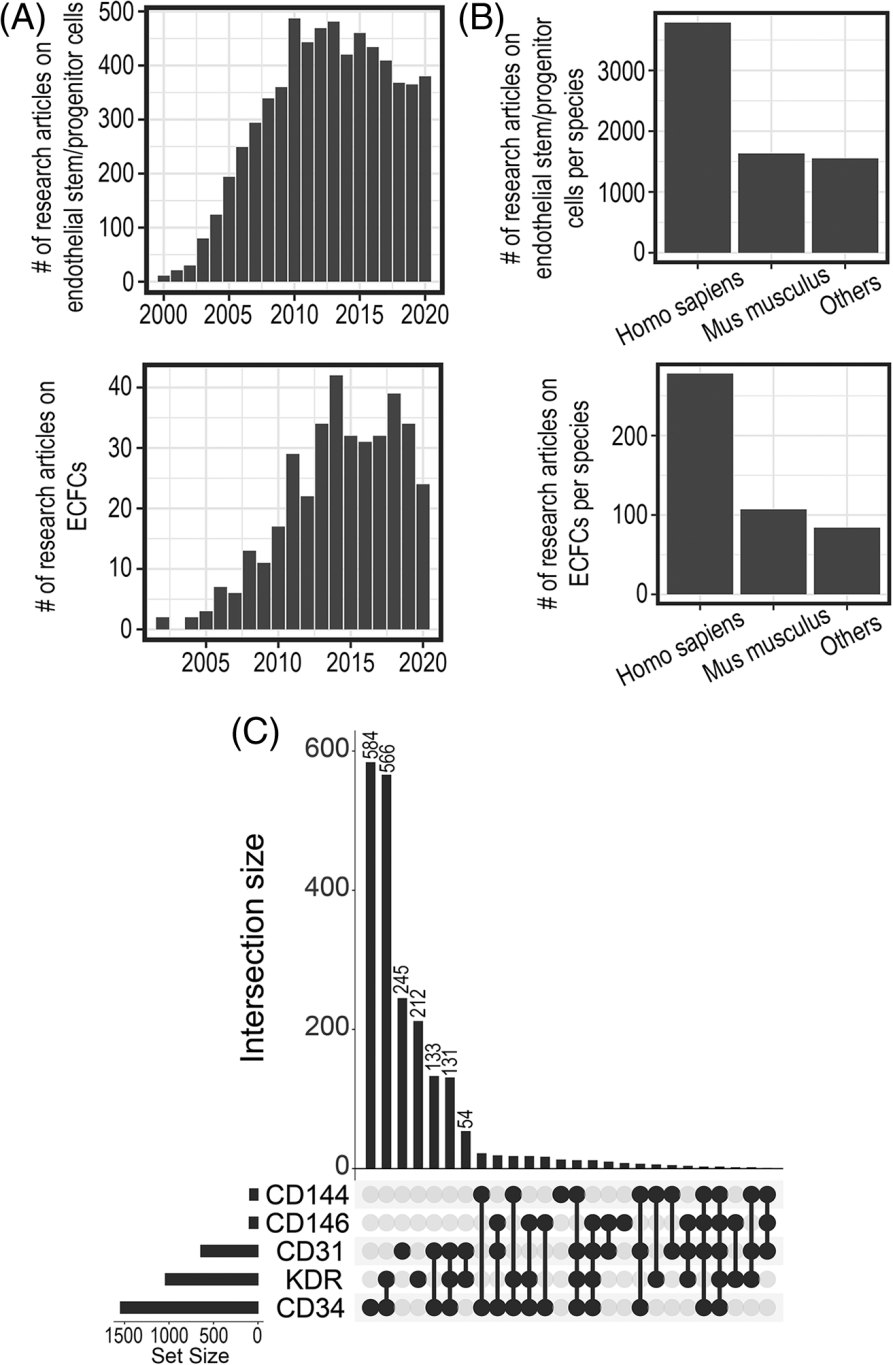
PubMed search for original research articles investigating endothelial stem/progenitor cells. A, Bar plot for the frequency of publications in endothelial stem/progenitor cells or ECFCs from 2000 to 2020. B, Bar plot with taxonomy distribution for papers identified. C, UpSet plot to depict frequently used molecular markers or combinations in studies for endothelial stem/progenitor cells

It is important to highlight that original findings demonstrating endothelial regeneration relied on mouse models. Similarly, endogenous vasoreparative cells have been described in various mouse organs. We have evaluated the scientific literature and performed a search in Pubmed using the query “endothelial progenitor” or “endothelial precursor” or “endothelial stem cell” or “endothelial colony‐forming cell” or “colony‐forming unit endothelial cell.” We further excluded review papers and finally identified 6437 non‐review articles published from 2000 to 2020. Within the same time frame of 20 years, we identified ~300 original research articles on ECFCs (Figure 2A). For each paper, we mined the metadata and collected the taxon id for the biological sample reported. As a result, we determined that 54% of original research papers on endothelial stem/progenitor cells are associated with human cells, 23% with murine cells and 23% with other organisms (Figure 2B). A similar trend was found for ECFC papers; ~60% associated with human cells and ~20% with mouse cells. These data showed that majority of studies relate to human cells; however, it is important to highlight that these investigations mostly used cells isolated in culture or quantified by flow cytometry. The studies in mouse models, on the other hand, have mostly focused on of endogenous endothelial stem cells and the characterization of potential molecular markers for such progenitors. In addition, differences between human and mouse endothelial progenitors have not been established. While there are similarities with some markers, phenotype, and functions, there is no data for a direct comparison among them; and we cannot assume these cell populations to be equivalent. Therefore, the human or mouse origin needs to be carefully considered when evaluating the literature, and more importantly, when using these data to support clinical trials.

## 4 MARKERS FOR ENDOTHELIAL PROGENITORS

Based on the original research articles published on “endothelial progenitors” collected from the last 20 years, we queried for molecular markers used to define cell identity. Among the most frequently used markers were CD34, VEGFR2, CD31, CD146, and CD144. CD34 alone appeared as the top cell marker in 584 out of 2119 papers. The second most common identity used was the combination of CD34 and VEGFR‐2, in 566 out of 2119 papers (Figure 2C). Enumeration of circulating endothelial progenitors in human blood is frequently assessed by flow cytometry for CD34 and VEGFR‐2 expression in mononucleated cells. This immunophenotypic definition for putative EPCs circulating in human peripheral blood has been often used for clinical studies, and these circulating CD34^+^VEGFR‐2^+^ cells are considered a measure of the endogenous reparative capacity of the cardiovascular system.^35^ A recent investigation on endothelial progenitor enumeration in blood from patients with COVID‐19 or undergoing bioprosthetic total artificial heart implantation, has employed mass cytometry and imaging flow cytometry to demonstrate that circulating CD19^−^CD34^+^ cells are negative for VEGFR‐2. Furthermore, VEGFR‐2 was only expressed in CD19^+^ B‐cells and CD14^+^ monocytes. Imaging cytometry showed that events which were CD19^−^CD34^+^VEGFR‐2^+^ represent cellular particles, fragments, or debris.^36^ These results have underscored the need to redefine circulating endothelial progenitors, in line with technological advances, such as mass cytometry, which increases accuracy for molecular definition of cells.

Alongside flow cytometry‐based enumeration of putative endothelial progenitors as CD34^+^VEGFR‐2^+^, cell culture‐based isolation of ECFCs is another well‐accepted way to study human endothelial progenitors. We summarize and discuss molecular markers that have been associated with resident endothelial stem/progenitors (Figure 1 and Supporting Information Table S1).

Additional markers, CD146 and CD144 have been suggested as substitutes for VEGFR‐2, or as additional markers to further refine the population.^37^ In addition, c‐Kit expression was associated with higher CD34 and VEGFR‐2 levels. CD34^+^c‐Kit^+^ have been proposed to be at the origin of endothelial recovery after total artificial heart transplantation.^36^ Added to this, c‐Kit expression has also been reported in quiescent endothelial stem cells residing in the vascular wall in mouse lung vasculature, expressing lin^−^CD31^+^CD105^+^Sca1^+^CD117(c‐kit)^+^.^38^ These cells showed in vitro colony forming capacity, and their regenerative potential was demonstrated by a single cell forming new blood vessels in vivo that connected with host circulation. Unfortunately, correlation between cytometry and cell culture isolation methods of endothelial progenitors has been difficult. Indeed, cultures of FACS‐sorted CD34^+^VEGFR2^+^ cells do not yield a comparable progenitor population to ECFCs generated by cell culture of unsorted PBMCs.^37^ Moreover, no correlation has been reported between the number of these circulating CD34^+^VEGFR2^+^ cells and the number of ECFCs obtained in cell culture.^39^ However, it has been suggested that it is the viable c‐Kit expressing cells in FACS‐sorted CD34^+^VEGFR‐2^+^ population that are the precursors to ECFCs in culture. Similarly, ECFCs were prospectively identified and isolated by expression of CD146 and lack of CD45 or CD133.^40^

Investigators have shown CD133 expression to be a marker of tissue‐resident endothelial progenitors in mouse lung.^41^ However, expression of CD133 as a marker of human derived ECFCs is controversial, and the ability of CD133^+^ cells to give rise to cells of endothelial lineage is highly debated.^42^, ^43^ Recently, Rossi et al sought to clear confusion around expression of CD133 in ECFCs, showing that human cord‐blood ECFCs do not express CD133 on their cell surface, but rather they express intracellular CD133; this is in direct contrast to mature ECs. Furthermore, silencing of CD133 causes a significant reduction in vasculogenic potential in a model of hindlimb ischaemia.^44^ This work has highlighted the potential for isolation of ECFCs by intracellular CD133 expression, and to further distinguish them from mature vascular ECs, of which their immunophenotype and tubulogenic capacity are highly similar^24^; although the exact mechanism of action for CD133 in ECFCs to enhance their vasculogenic potential remains unknown. In line with this, stem cells from infantile hemangiomas (Hem‐SC) isolated as CD133^+^ cells, are a mesenchymal population capable of differentiating into endothelial cells.^45^

VEGFR‐2 co‐receptor, NRP1, has been identified as a marker of an endothelial precursor, since its expression precedes that of CD31 and CD34 in mouse embryonic studies.^46^ Following on from this, a protocol was developed to generate ECFC‐like cells from induced pluripotent stem cells that were directly comparable to cord‐blood derived ECFCs in vasculogenic potential. During the differentiation process, cells were sorted based upon NRP1^+^CD31^+^ expression, and when expanded demonstrated high clonal proliferative potential and in vivo vessel formation that inosculated with host vasculature in the hindlimb ischemia model and oxygen induced retinopathy model.^47^ As such, iPS‐derived ECFCs could represent a robust and patient‐specific source of ECFCs for cell therapy trials. Alternative approaches for rapid, consistent, and highly efficient differentiation of iPSCs into endothelial cells include the timely activation of transcription factor ETV2.^48^, ^49^ However, more research into safety and memory of host susceptibility to disease is important to make this cell therapy a reality.

The glycoprotein von Willebrand factor (VWF) is frequently used as an endothelial cell marker and its expression increases during iPS to endothelial cell differentiation. In addition, VWF regulates blood vessel formation.^50^ VWF knockdown in ECFCs led to increased proliferation, migration, and in vitro angiogenesis.^51^ The considerable heterogeneity in VWF expression in mature endothelial cells and ECFCs suggests population heterogeneity and VWF expression dynamics between progenitors and differentiated cells remains to be elucidated.

IPS‐derived endothelial cells can be differentiated by various methods, including embryoid body formation or cell culture with/without feeder layers. Studies have shown that differentiation toward an endothelial lineage is dependent on certain pathways (eg, WNT, TGFβ, BMP, and VEGF signaling) or differential cues from the microenvironment.^52^ Since IPSCs are pluripotent cells, in vitro models for endothelial cell differentiation and use of new technologies, such as scRNAseq, will enable the characterization of cell intermediates between iPS cells, endothelial stem cells and differentiated endothelial cells, at the transcriptional level. This would not only improve efficiency of differentiation protocols, but also aid understanding of vascular development.

There are some recent reports characterizing new markers to identify endothelial vasoreparative cells in the mouse. CD157 has been identified as a marker for tissue‐resident vascular endothelial stem cells in numerous mouse organs. CD157^+^ cells are enriched in the liver endothelial side population, and importantly, a single CD157^+^CD200^+^ endothelial cell was capable of repairing damaged liver vasculature by differentiating into progeny within large and small vessels. Similarly, transplantation of CD157^+^CD200^+^ endothelial cells engrafted into mouse vasculature and corrected the bleeding phenotype in hemophilia A mice.^31^ Murine vascular endothelial stem cells (CD45^−^CD31^+^CD157^+^) demonstrated enhanced endothelial tubule network formation, and exhibited clonal endothelial colony forming capacity vs the CD157^−^ population.^53^ In this protocol, ECFCs were supported using an OP9 stromal cell coculture approach as previously described.^54^ Although these results are akin to human ECFCs, their existence in humans has not yet been proven.^31^ Nevertheless, CD157 has recently been used to identify ECFCs with highly proliferative potential, generated following differentiation from human pluripotent stem cells (hPSCs). CD157 expression was significantly higher in hPSCs‐derived ECFCs (46%) compared with mature human saphenous vein endothelial cells (2%); however, functional readouts of this CD157^+^ high proliferative potential (HPP)‐ECFC population in humans is warranted.^55^

Endothelial protein C receptor (EPCR) is a glycoprotein with anti‐thrombotic and cytoprotective roles. EPCR expressing endothelial cells have also been suggested as vascular endothelial stem cells. EPCR^+^ endothelial cells showed in vitro clonogenicity by serial passage for at least 10 passages in more than 3 months. Long‐term lineage tracing demonstrated that EPCR^+^ cells contribute to endothelial cell expansion during development of mammary vasculature, at 10 months post activation of the GFP label. Furthermore, EPCR^+^ cells were shown to have differentiation capacity into pericytes and exhibited endothelial‐to‐mesenchymal transition (EndMT) molecular signatures which included upregulation of Zeb1, Zeb2, Foxc2, and Vimentin. EndMT is defined as a phenotypic switch where endothelial cells lose their endothelial characteristics and acquire mesenchymal traits. It has been suggested that some level of EndMT is required to initiate angiogenesis by endothelial sprouting. Genetic ablation of EPCR^+^ cells severely delayed vascular development in mouse retinas, and EPCR^+^ cells delivered into the mouse hindlimb ischemia model improved blood flow with effective engraftment.^56^ However, in vitro studies using mature human endothelial cells (HRECs, HAECs, HUVECs) also show high cell surface expression of EPCR.^57^ Potentially, EPCR^+^ vascular endothelial stem cells are mouse‐specific, or expression of EPCR in human ECs has become enriched during in vitro cell culture compared with in situ. In addition, studies have shown that expression of EPCR marks long‐term hematopoietic stem cells within mouse bone marrow^58^ and human cord blood^59^; therefore, it is important to include an endothelial specific marker to ensure definitive expression of EPCR in endothelial cells.

Endogenous endothelial stem cells in the mouse aorta have been defined as CD31^low^ VEGFR2^−/low^, which give rise to transit amplifying CD31^int^VEGFR‐2^−/low^ and definitive differentiated CD31^high^VEGFR‐2^high^ cells (Figure 1). These endothelial stem cells were also characterized by the expression of IL33 and Sox9.^29^ Furthermore, CD31^low^VEGFR‐2^−^IL33^+^Sox9^+^ endothelial stem cells in the aorta exhibited high expression of Pdgfrα, which suggested the existence of a bipotent meso‐endothelial progenitor. This was confirmed in human placenta.^60^ When expression of CD157 and EPCR (PROCR) were assessed in the scRNAseq data from tissue resident endothelial stem cells in the mouse aorta, these markers could not distinguish endothelial cell subsets.^30^ This suggests that endothelial stem cell characteristics may be organ specific. Interestingly, scRNA‐seq and lineage tracing experiments have also revealed a transcriptional hierarchy in resident endothelial cells from the mouse heart.^61^ When a myocardial infarction model was investigated, a subset of resident endothelial cells expressing Plvap was associated with neovasculogenesis. Plvap is a protein responsible for vascular fenestrae and caveolae diaphragms, which modulates vascular permeability; however, its association with neovasculogenesis in ischemic hearts suggests that high Plvap expression may also denote a resident endogenous vasoreparative cell population. Plvap expression was also found increased in ischemic human hearts.

Characterization of side population cells in the mouse lung identified CD45^−^CD31^+^VEGFR‐2^−^ cells as progenitors capable of differentiating into smooth muscle cells and endothelial cells.^62^ In vitro studies determined that CD45^−^CD31^+^VEGFR‐2^−^ cells differentiate into CD45^−^CD31^+^VEGFR‐2^+^ cells which give rise to endothelial cells; suggesting VEGFR‐2 expression as a sign of late endothelial progenitor commitment. The side population is characterized by their ability to efflux fluorescent dyes via ATP‐binding transporters such as ABCG2; and a recent study on lung resident ABCG2^+^ cells has identified them as mesenchymal vascular progenitors with capacity to modulate adaptive repair angiogenesis in the lung.^63^

## 5 CONCLUSION

Stem cell biology is being transformed thanks to technological advances such as single cell RNA sequencing, mass cytometry, imaging cytometry, and cell‐fate mapping microscopy. Use of these technologies has revealed shortcomings and inaccuracies in previous approaches to define and locate endothelial progenitors. Here, we emphasized that the endothelial phenotype, coupled with self‐renewal and differentiation capacities, must be essential features to define an endothelial stem cell. We also highlight that bone marrow can no longer be accepted as the sole source for isolation of endothelial stem/progenitor cells, as there is evidence to support a vascular niche. Careful attention should be drawn to research using mouse vs human cells, as some results may not be easily replicated. For example, while isolation of human ECFCs is optimized, most labs find the isolation of mouse ECFCs challenging. Lastly, various surface markers such as CD157 or EPCR have been proposed to identify tissue‐resident endothelial stem cells, but more research is warranted to establish overlap and relationships among these markers. The absence of a robust and exhaustive marker to clearly identify endothelial progenitors alongside the inherent species‐species variation and the impact of the disease on the cellular properties, highlights the need to define these cells using a profile of combination of markers, rather than a single marker, coupled with appropriate functional assays to establish an operational definition of the endothelial cell differentiation hierarchy.

## CONFLICT OF INTEREST

The authors declared no potential conflicts of interest.

## AUTHOR CONTRIBUTIONS

S.E.J.C., V.P., E.P.: data collection and assembly, data analysis and interpretation, manuscript writing, final approval of manuscript; L.S., N.G., C.L.G.: data interpretation, manuscript writing, final approval of manuscript; A.W.S., D.M.S., R.J.M.: conception and design, data analysis and interpretation, manuscript writing, final approval of manuscript.

## Supporting information


**Table S1** Supporting information

## Data Availability

Data sharing is not applicable to this article as no new data were created or analyzed in this study.

## References

[1] Qiu J , Hirschi KK . Endothelial cell development and its application to regenerative medicine. Circ Res. 2019;125:489‐501. 31518171 10.1161/CIRCRESAHA.119.311405 PMC8109152

[2] Coşxkun S , Chao H , Vasavada H , et al. Development of the fetal bone marrow niche and regulation of HSC quiescence and homing ability by emerging osteolineage cells. Cell Rep. 2014;9:581‐590. 25310984 10.1016/j.celrep.2014.09.013 PMC4266564

[3] Lacaud G , Kouskoff V . Hemangioblast, hemogenic endothelium, and primitive versus definitive hematopoiesis. Exp Hematol. 2017;49:19‐24. 28043822 10.1016/j.exphem.2016.12.009

[4] Loges S , Fehse B , Brockmann MA , et al. Identification of the adult human hemangioblast. Stem Cells Dev. 2004;13:229‐242. 15186719 10.1089/154732804323099163

[5] Grant MB , May WS , Caballero S , et al. Adult hematopoietic stem cells provide functional hemangioblast activity during retinal neovascularization. Nat Med. 2002;8:607‐612. 12042812 10.1038/nm0602-607

[6] Rigato M , Monami M , Fadini GP . Autologous cell therapy for peripheral arterial disease. Circ Res. 2017;120:1326‐1340. 28096194 10.1161/CIRCRESAHA.116.309045

[7] Medina RJ , O'Neill CL , O'Doherty TM , et al. Myeloid angiogenic cells act as alternative M2 macrophages and modulate angiogenesis through interleukin‐8. Mol Med. 2011;17:1045‐1055. 21670847 10.2119/molmed.2011.00129 PMC3188859

[8] Prokopi M , Pula G , Mayr U , et al. Proteomic analysis reveals presence of platelet microparticles in endothelial progenitor cell cultures. Blood. 2009;114:723‐732. 19369228 10.1182/blood-2009-02-205930

[9] Bergmann O , Zdunek S , Felker A , et al. Dynamics of cell generation and turnover in the human heart. Cell. 2015;161:1566‐1575. 26073943 10.1016/j.cell.2015.05.026

[10] Arrojo e Drigo R , Lev‐Ram V , Tyagi S , et al. Age Mosaicism across multiple scales in adult tissues. Cell Metab. 2019;30:343‐351.e3. 31178361 10.1016/j.cmet.2019.05.010 PMC7289515

[11] Chen Q , Liu Y , Jeong HW , et al. Apelin+ endothelial niche cells control hematopoiesis and mediate vascular regeneration after Myeloablative injury. Cell Stem Cell. 2019;25:768‐783.e6. 31761723 10.1016/j.stem.2019.10.006 PMC6900750

[12] Nagao RJ , Fortin CL , Stevens KR , et al. Human organ‐specific endothelial cell heterogeneity. IScience. 2018;4:20‐35. 30240741 10.1016/j.isci.2018.05.003 PMC6147238

[13] Cleuren ACA , van der Ent MA , Jiang H , et al. The in vivo endothelial cell translatome is highly heterogeneous across vascular beds. Proc Natl Acad Sci USA. 2019;116:23618‐23624. 31712416 10.1073/pnas.1912409116 PMC6876253

[14] Rafii S , Butler JM , Sen DB . Angiocrine functions of organ‐specific endothelial cells. Nature. 2016;529:316‐325. 26791722 10.1038/nature17040 PMC4878406

[15] Borges L , Oliveira VKP , Baik J , Bendall SC , Perlingeiro RCR . Serial transplantation reveals a critical role for endoglin in hematopoietic stem cell quiescence. Blood. 2019;133:688‐696. 30593445 10.1182/blood-2018-09-874677 PMC6376279

[16] Banno K , Yoder MC . Tissue regeneration using endothelial colony‐forming cells: promising cells for vascular repair. Pediatr Res. 2018;83:283‐290. 28915234 10.1038/pr.2017.231

[17] Medina RJ , Barber CL , Sabatier F , et al. Endothelial progenitors: a consensus statement on nomenclature. Stem Cells Translational Medicine. 2017;6:1316‐1320. 28296182 10.1002/sctm.16-0360 PMC5442722

[18] O'Neill CL , McLoughlin KJ , Chambers SEJ , et al. The vasoreparative potential of endothelial colony forming cells: a journey through pre‐clinical studies. Front Med. 2018;5:273. 10.3389/fmed.2018.00273 PMC6232760 30460233

[19] Reid E , Guduric‐Fuchs J , O'Neill CL , et al. Preclinical evaluation and optimization of a cell therapy using human cord blood‐derived endothelial Colony‐forming cells for ischemic retinopathies. Stem Cells Translational Medicine. 2017;7(1):59–67. 29164803 10.1002/sctm.17-0187 PMC5746158

[20] Liao G , Zheng K , Shorr R , Allan DS . Human endothelial colony‐forming cells in regenerative therapy: a systematic review of controlled preclinical animal studies. Stem Cells Translational Medicine. 2020;9:1344‐1352. 32681814 10.1002/sctm.20-0141 PMC7581447

[21] Lin Y , Weisdorf DJ , Solovey A , Hebbel RP . Origins of circulating endothelial cells and endothelial outgrowth from blood. J Clin Invest. 2000;105:71‐77. 10619863 10.1172/JCI8071 PMC382587

[22] Fujisawa T , Tura‐Ceide O , Hunter A , et al. Endothelial progenitor cells do not originate from the bone marrow. Circulation. 2019;140:1524‐1526. 31657952 10.1161/CIRCULATIONAHA.119.042351 PMC6818974

[23] Green L , Ofstein RH , Rapp B , et al. Adult venous endothelium is a niche for highly proliferative and vasculogenic endothelial colony‐forming cells. J Vasc Surg. 2017;66:1854‐1863. 28655551 10.1016/j.jvs.2016.11.059

[24] Kutikhin AG , Tupikin AE , Matveeva VG , et al. Human peripheral blood‐derived endothelial colony‐forming cells are highly similar to mature vascular endothelial cells yet demonstrate a transitional transcriptomic signature. Cell. 2020;9:876. 10.3390/cells9040876 PMC7226818 32260159

[25] Solomon I , O'Reilly M , Ionescu L , et al. Functional differences between placental micro‐ and macrovascular endothelial colony‐forming cells. Stem Cells Translational Medicine. 2016;5:291‐300. 26819255 10.5966/sctm.2014-0162 PMC4807658

[26] Lin RZ , Moreno‐Luna R , Muñoz‐Hernandez R , et al. Human white adipose tissue vasculature contains endothelial colony‐forming cells with robust in vivo vasculogenic potential. Angiogenesis. 2013;16:735‐744. 23636611 10.1007/s10456-013-9350-0 PMC3762916

[27] Kawasaki T , Nishiwaki T , Sekine A , et al. Vascular repair by tissue‐resident endothelial progenitor cells in endotoxin‐induced lung injury. Am J Respir Cell Mol Biol. 2015;53:500‐512. 25719275 10.1165/rcmb.2014-0185OC

[28] McDonald AI , Shirali AS , Aragón R , et al. Endothelial regeneration of large vessels is a biphasic process driven by local cells with distinct proliferative capacities. Cell Stem Cell. 2018;23:210‐225.e6. 30075129 10.1016/j.stem.2018.07.011 PMC6178982

[29] Patel J , Seppanen EJ , Rodero MP , et al. Functional definition of progenitors versus mature endothelial cells reveals key SoxF‐dependent differentiation process. Circulation. 2017;135:786‐805. 27899395 10.1161/CIRCULATIONAHA.116.024754

[30] Lukowski SW , Patel J , Andersen SB , et al. Single‐cell transcriptional profiling of aortic endothelium identifies a hierarchy from endovascular progenitors to differentiated cells. Cell Rep. 2019;27:2748‐2758.e3. 31141696 10.1016/j.celrep.2019.04.102

[31] Wakabayashi T , Naito H , Ichi SJ , et al. CD157 marks tissue‐resident endothelial stem cells with homeostatic and regenerative properties. Cell Stem Cell. 2018;22:384‐397.e6. 29429943 10.1016/j.stem.2018.01.010

[32] Meng S , Lv J , Chanda PK , Owusu I , Chen K , Cooke JP . Reservoir of fibroblasts promotes recovery from limb ischemia. Circulation. 2020;142:1647‐1662. 32820662 10.1161/CIRCULATIONAHA.120.046872 PMC7987209

[33] He L , Huang X , Kanisicak O , et al. Preexisting endothelial cells mediate cardiac neovascularization after injury. J Clin Invest. 2017;127:2968‐2981. 28650345 10.1172/JCI93868 PMC5531398

[34] Fisher SA , Doree C , Mathur A , et al. Cochrane corner: stem cell therapy for chronic ischaemic heart disease and congestive heart failure. Heart. 2018;104(1):8‐10. 28607164 10.1136/heartjnl-2017-311684

[35] Fadini GP , Mehta A , Dhindsa DS , et al. Circulating stem cells and cardiovascular outcomes: from basic science to the clinic. Eur Heart J. 2020;41(44):4271‐4282. 31891403 10.1093/eurheartj/ehz923 PMC7825095

[36] Guerin CL , Guyonnet L , Goudot G , et al. Multidimensional proteomic approach of endothelial progenitors demonstrate expression of KDR restricted to CD19 cells. Stem Cell Rev Rep. 2021;17(2):639‐651. 33205351 10.1007/s12015-020-10062-1 PMC7670993

[37] Huizer K , Mustafa DAM , Spelt JC , Kros JM , Sacchetti A . Improving the characterization of endothelial progenitor cell subsets by an optimized FACS protocol. PLoS One. 2017;12:e0184895. 28910385 10.1371/journal.pone.0184895 PMC5599045

[38] Fang S , Wei J , Pentinmikko N , Leinonen H , Salven P . Generation of functional blood vessels from a single c‐kit+ adult vascular endothelial stem cell. PLoS Biol. 2012;10:e1001407. 23091420 10.1371/journal.pbio.1001407 PMC3473016

[39] Smadja DM , Mauge L , Nunes H , et al. Imbalance of circulating endothelial cells and progenitors in idiopathic pulmonary fibrosis. Angiogenesis. 2013;16:147‐157. 22983452 10.1007/s10456-012-9306-9

[40] Mund JA , Estes ML , Yoder MC , Ingram DA Jr , Case J . Flow cytometric identification and functional characterization of immature and mature circulating endothelial cells. Arterioscler Thromb Vasc Biol. 2012;32:1045‐1053. 22282356 10.1161/ATVBAHA.111.244210 PMC3306529

[41] Sekine A , Nishiwaki T , Nishimura R , et al. Prominin‐1/CD133 expression as potential tissue‐resident vascular endothelial progenitor cells in the pulmonary circulation. Am J Physiol—Lung Cell Mol Physiol. 2016;310:L1130‐L1142. 27059286 10.1152/ajplung.00375.2014

[42] Case J , Mead LE , Bessler WK , et al. Human CD34+AC133+VEGFR‐2+ cells are not endothelial progenitor cells but distinct, primitive hematopoietic progenitors. Exp Hematol. 2007;35:1109‐1118. 17588480 10.1016/j.exphem.2007.04.002

[43] Timmermans F , Van Hauwermeiren F , De Smedt M , et al. Endothelial outgrowth cells are not derived from CD133+ cells or CD45+ hematopoietic precursors. Arterioscler Thromb Vasc Biol. 2007;27:1572‐1579. 17495235 10.1161/ATVBAHA.107.144972

[44] Rossi E , Poirault‐Chassac S , Bieche I , et al. Human endothelial Colony forming cells express intracellular CD133 that modulates their Vasculogenic properties. Stem Cell Rev Rep. 2019;15:590‐600. 30879244 10.1007/s12015-019-09881-8

[45] Boscolo E , Mulliken JB , Bischoff J . VEGFR‐1 mediates endothelial differentiation and formation of blood vessels in a murine model of infantile hemangioma. Am J Pathol. 2011;179:2266‐2277. 21945324 10.1016/j.ajpath.2011.07.040 PMC3204018

[46] Cimato T , Beers J , Ding S , et al. Neuropilin‐1 identifies endothelial precursors in human and murine embryonic stem cells before cd34 expression. Circulation. 2009;119:2170‐2178. 19364973 10.1161/CIRCULATIONAHA.109.849596 PMC2774135

[47] Prasain N , Lee MR , Vemula S , et al. Differentiation of human pluripotent stem cells to cells similar to cord‐blood endothelial colony‐forming cells. Nat Biotechnol. 2014;32:1151‐1157. 25306246 10.1038/nbt.3048 PMC4318247

[48] Ng AHM , Khoshakhlagh P , Rojo Arias JE , et al. A comprehensive library of human transcription factors for cell fate engineering. Nat Biotechnol. 2021;39(4):510‐519. 33257861 10.1038/s41587-020-0742-6 PMC7610615

[49] Wang K , Lin RZ , Hong X , et al. Robust differentiation of human pluripotent stem cells into endothelial cells via temporal modulation of ETV2 with modified mRNA. Sci Adv. 2020;6(30):eaba7606. 32832668 10.1126/sciadv.aba7606 PMC7439318

[50] Randi AM , Smith KE , Castaman G . Von Willebrand factor regulation of blood vessel formation. Blood. 2018;132:132‐140. 29866817 10.1182/blood-2018-01-769018 PMC6182264

[51] Starke RD , Ferraro F , Paschalaki KE , et al. Endothelial von Willebrand factor regulates angiogenesis. Blood. 2011;117:1071‐1080. 21048155 10.1182/blood-2010-01-264507 PMC3035068

[52] Williams IM , Wu JC . Generation of endothelial cells from human pluripotent stem cells methods, considerations, and applications. Arterioscler Thromb Vasc Biol. 2019;39:1317‐1329. 31242035 10.1161/ATVBAHA.119.312265 PMC6597190

[53] Naito H , Wakabayashi T , Ishida M , et al. Isolation of tissue‐resident vascular endothelial stem cells from mouse liver. Nat Protoc. 2020;15:1066‐1081. 32005982 10.1038/s41596-019-0276-x

[54] Lin Y , Gil CH , Yoder MC . Identification of circulating endothelial colony‐forming cells from murine embryonic peripheral blood. Methods Mol Biol. 2019;1940:97‐107. 30788820 10.1007/978-1-4939-9086-3_7

[55] Farkas S , Simara P , Rehakova D , Veverkova L , Koutna I . Endothelial progenitor cells produced from human pluripotent stem cells by a synergistic combination of cytokines, small compounds, and serum‐free medium. Front Cell Dev Biol. 2020;8(309). 10.3389/fcell.2020.00309 PMC7249886 32509776

[56] Yu QC , Song W , Wang D , Zeng YA . Identification of blood vascular endothelial stem cells by the expression of protein C receptor. Cell Res. 2016;26:1079‐1098. 27364685 10.1038/cr.2016.85 PMC5113308

[57] Arta A , Eriksen AZ , Melander F , et al. Endothelial protein C‐targeting liposomes show enhanced uptake and improved therapeutic efficacy in human retinal endothelial cells. Investig Ophthalmol Vis Sci. 2018;59:2119‐2132. 29677376 10.1167/iovs.18-23800

[58] Rabe JL , Hernandez G , Chavez JS , et al. CD34 and EPCR coordinately enrich functional murine hematopoietic stem cells under normal and inflammatory conditions. Exp Hematol. 2020;81:1‐15.e6. 31863798 10.1016/j.exphem.2019.12.003 PMC6938677

[59] Fares I , Chagraoui J , Lehnertz B , et al. EPCR expression marks UM171‐expanded CD34+ cord blood stem cells. Blood. 2017;129:3344‐3351. 28408459 10.1182/blood-2016-11-750729

[60] Shafiee A , Patel J , Hutmacher DW , Fisk NM , Khosrotehrani K . Meso‐endothelial bipotent progenitors from human placenta display distinct molecular and cellular identity. Stem Cell Rep. 2018;10:890‐904. 10.1016/j.stemcr.2018.01.011 PMC5918195 29478891

[61] Li Z , Solomonidis EG , Meloni M , et al. Single‐cell transcriptome analyses reveal novel targets modulating cardiac neovascularization by resident endothelial cells following myocardial infarction. Eur Heart J. 2019;40:2507‐2520. 31162546 10.1093/eurheartj/ehz305 PMC6685329

[62] Xu Y , Sun P , Wang JY , et al. Differentiation of CD45−/CD31+ lung side population cells into endothelial and smooth muscle cells in vitro. Int J Mol Med. 2019;43:1128‐1138. 30628669 10.3892/ijmm.2019.4053 PMC6365051

[63] Summers ME , Richmond BW , Menon S , et al. Resident mesenchymal vascular progenitors modulate adaptive angiogenesis and pulmonary remodeling via regulation of canonical Wnt signaling. FASEB J. 2020;34:10267‐10285. 32533805 10.1096/fj.202000629R PMC7496763

