# Supplementary material for: Current concepts on endothelial stem cells definition, location, and markers
Source: Stem Cells Transl Med. 2021 Nov 1;10(Suppl 2):S54–61. doi: 10.1002/sctm.21-0022 (PMC8560200; doi:10.1002/sctm.21-0022)
Supplement: Supplementary file 1 — Table S1 Supporting information [file SCT3-10-S54-s001.pdf]

Supplementary Table 1. Summary of molecular markers used to identify endothelial stem/progenitor cells

Marker	Species	Origin	Isolation Protocol		Notes	PMID
			Sorting	Culture		
**CD34+ VEGFR2+**	Human	Cord blood, peripheral blood	CD45- CD34+ VEGFR2+	N/A	CD146 and CD144 further define the population	28910385
**C-Kit+**	Mouse	Lung vasculature	Lin-CD31+ CD105+Sca1+ CD117(ckit)+	Produce tens of millions of daughter endothelial cells in vitro	Generate in vivo functional blood vessels	23091420
**CD133+**	Mouse	Lung vasculature	CD31+CD133+	Culture of sorted cells gave rise to colonies that formed fine vascular networks	Tissue resident endothelial progenitor	27059286
	Human	Cord blood, Bone marrow	CD34+AC133+ VEGFR2+	Culture of sorted cells did not yield endothelial progeny	CD133+ are hematopoietic precursors	17588480
			CD34+CD45-	Generated cells express VEGFR2, not CD133		17495235
		Cord blood	N/A	Intracellular CD133 expression	Silencing of CD133 abolished post-ischaemia neovascularization	30879244
		Infantile haemangio mas	CD133+	Single CD133+ cells expanded as clonal populations that expressed CD31	Selected CD31 cells formed blood vessels in vivo	18535669
		Bone marrow	Small CD133+ Lin-CD45- (VSELs)	Expand in culture and differentiate into cells from different germ layers, including ECs.	A potential competitor for ESCs and iPSCs. Angiogenic properties in HLI model	25608764
**NRP1+**	Human	Induced Pluripotent Stem Cells	Differentiated towards endothelial lineage in 12day protocol, then sorted for NRP1+CD31+	Sorted cells had high clonal proliferative potential. Directly comparable to CB-ECFCs	In vivo vessel forming ability in model of HLI and OIR	25306246
**CXCR4+**	Human	Peripheral blood	N/A	Cells expressed more CXCR4 than HUVECs	CXCR4 is marker of tip cell activity	26113473

Supplementary Table 1. Summary of molecular markers used to identify endothelial stem/progenitor cells

**CD157+**	Mouse	Liver	CD31+VEcad+ CD157+CD200+	CD157+CD200+ECs formed larger colonies with higher efficiency than CD157-CD200- and CD157-CD200-	Long term lineage tracing shows contribution of CD157+ ECs to maintenance of blood vessels long term	29429943
		Liver, Lung, Limb muscle, Small intestine, Pancreas	CD45-CD31+ CD157+	EC network formation and colony formation higher in CD157+ vs CD157- population		32005982
	Human	Pluripotent stem cells	N/A	Differentiated towards endothelial lineage in 5day protocol	iPS-derived endothelial progenitors expressed CD157 highly, in addition to CD31, CD144, CD34, VEGFR2, and CXCR4	32509776
**EPCR+**	Mouse	Mammary fat pad	Lin-CD31+ CD105+EPCR+	In vitro clonogenicity by serial passage for at least 10 passages	EPCR+ selection enriched for vascular endothelial stem cells. Potential to differentiate into endothelial cells and pericytes	27364685
**CD31low VEGFR2- /low IL33+ SOX9+**	Mouse	Aorta	CD34+CD45- CD31^{low} VEGFR2$^{-/low}$	In vitro colony forming ability and self-renewal capacity in vivo	Defined a novel endothelial hierarchy as CD31^{low}VEGFR2$^{-/low}$ to CD31^{int}VEGFR2$^{-/low}$ to CD31^{hi}VEGFR2^{hi}	27899395
**CD45- side population**	Mouse	Embryonic Lung	CD45-CD31+	Express gene characteristics of endothelium, but do not grow or differentiate in vitro by themselves	Co-cultures with smooth muscle progenitors (CD45-CD31-) formed tube-like structures in vitro	15802552
		Adult Lung	CD45-CD31+ VEGFR2-	Formed colonies in vitro and differentiated into CD45-CD31+VEGFR2+ cells which then differentiated into ECs	CD45-CD31+VEGFR2- could also yield smooth muscle progenitors	30628669
